# Plasma DNA and deoxyribonuclease are associated with glucose metabolism in healthy mice

**DOI:** 10.1371/journal.pone.0265099

**Published:** 2022-04-06

**Authors:** Katarína Kmeťová, Jozef Čonka, Jakub Janko, Júlia Illés, Oľga Uličná, Peter Celec

**Affiliations:** 1 Faculty of Medicine, Institute of Molecular Biomedicine, Comenius University, Bratislava, Slovakia; 2 Faculty of Medicine, Pharmacobiochemical Laboratory of Third Department of Internal Medicine, Comenius University, Bratislava, Slovakia; 3 Faculty of Medicine, Institute of Pathophysiology, Comenius University, Bratislava, Slovakia; 4 Faculty of Natural Sciences, Department of Molecular Biology, Comenius University, Bratislava, Slovakia; CHA University, REPUBLIC OF KOREA

## Abstract

It is currently unknown why obesity leads in some patients to prediabetes and metabolic syndrome. Microinflammation potentially caused by extracellular DNA is supposed to be involved. The aim of this cross-sectional study in healthy mice was to analyze the association between plasma extracellular DNA and glucose metabolism. Fasting glycemia and insulin were measured in healthy adult female mice that subsequently underwent an oral glucose tolerance test. Indices of glucose metabolism and insulin sensitivity were calculated. DNA was isolated from plasma and quantified fluorometrically. Deoxyribonuclease (DNase) activity of plasma was measured using the single radial enzyme diffusion method. Fasting glycemia correlated negatively with both, extracellular DNA and DNase (r = -0.44 and r = -0.32, respectively). DNase was associated positively with the incremental area under curve (r = 0.35), while extracellular DNA correlated negatively with total area under curve of glycemia during oral glucose tolerance test (r = -0.34). Measures of insulin sensitivity were found to be associated with neither extracellular DNA, nor DNase. The hypothesis of an association of low DNase with increased fasting glucose was partially proved. Surprisingly, low extracellular DNA is associated with higher fasting glucose and lower glucose tolerance in mice. As novel therapeutic targets for prediabetes and metabolic syndrome are highly needed, this study provides novel unexpected associations within the limitations of the focus on physiological variability as it was conducted on healthy mice. The causality of these associations should be proved in further interventional experiments.

## Introduction

Obesity is often, but not always, associated with the metabolic syndrome [1]. The pathogenesis that leads to the presence of insulin resistance, hyperlipidemia and several other components of the syndrome is not clear, but likely includes microinflammation [2]. However, the cause of the inflammation is unknown, sustainable weight loss is difficult to achieve, and the clinical consequence is that there is no causal and effective treatment available for patients with metabolic syndrome. Identification of the cause of metabolic complications of obesity could, thus, provide a new therapeutic target for a large patient population [3].

Dying cells passively or actively release their DNA into the extracellular space [4]. The so-called cell-free or extracellular DNA (ecDNA) has been overlooked for decades until it was rediscovered as a highly potential source of information for non-invasive prenatal diagnosis and cancer screening [5–7]. However, ecDNA is not only a promising biomarker, but also a biologically active molecule that is recognized by immune cells via DNA receptors [8]. The subsequent activation of the immune cells might lead to inflammation that is involved in various diseases such as systematic lupus, rheumatoid arthritis and other rheumatoid diseases [9], but also in hepatorenal injury [10] and sepsis [11].

Previous studies have already shown that ecDNA is increased in obese women and female mice [12,13]. This ecDNA might come from dying adipocytes, but also from activated neutrophils and macrophages that are able to produce extracellular traps from their DNA as part of the inflammatory response [14,15]. In an experimental study Nishimoto and colleagues have proved that ecDNA recognized by the Toll-like receptor 9 is involved in metabolic complications of obesity, at least in mice [16]. Genetic deficiency of Toll like receptor 9 protected mice from insulin resistance in diet-induced obesity. Similarly, it has been shown that higher concentrations of mitochondrial DNA in plasma is associated with higher insulin resistance in mice [17] and also in patients with type 2 diabetes [18]. Thus, it seems that plasma ecDNA concentrations are related to insulin sensitivity/resistance both, in patients and in disease models, but it is unclear whether this is also true for healthy population.

The published findings suggest that ecDNA could be responsible for the microinflammation in metabolic complications of obesity [15,16]. However, whether interindividual variability of ecDNA is associated with physiological variability of the glucose metabolism is unknown. This potential association is not intriguing especially, because there is already a potential therapeutic/preventive intervention–the administration of exogenous deoxyribonuclease (DNase) that cleaves ecDNA [19]. In our previous study, we showed that there is greater interindividual variability in ecDNA in female mice comparing to males [20], therefore, this study primarily aimed at female mice. Besides exogenous DNase, there is also endogenous DNase that could be a factor explaining the biological variability of the metabolism [21].

The aim of our study was to analyze the variability of plasma ecDNA and DNase activity in association with biochemical parameters of glucose metabolism and insulin sensitivity in healthy adult female mice. We hypothesized that high ecDNA and low DNase will be associated with high fasting glucose and insulin, as well as with impaired glucose tolerance.

## Methods

### Animal housing

Healthy adult female mice (n = 78) of the outbred CD-1 strain (Velaz, Prague, Czech Republic) were housed in conventional cages under standard conditions (21–24°C environmental temperature and 55–65% humidity) with a 12/12 h light-dark cycle and had ad libitum access to food and drink. Mice were fed with standard chow (Ssniff R/M-H, Spezialdiäten GmbH, Soest, Germany). At the age of 11 weeks, mice underwent an oral glucose tolerance test (oGTT) and were sacrificed. All of the methods and procedures were conducted in accordance with the EU Directive 2010/63/EU and Slovak legislation. All experimental protocols were approved by the Ethics Committee of the Institute of Molecular Biomedicine, Comenius University, Bratislava.

### Experimental design

Healthy female mice were used for a cross-sectional study without any intervention. The mice underwent glucose metabolism evaluation: fasting blood collection for glucose and insulin measurement and oGTT. Mice were sacrificed, terminal blood was collected for analysis of ecDNA and DNase activity, liver was collected for determination of triacylglycerols and cholesterol to analyze liver damage.

### Oral glucose tolerance test and insulin sensitivity measurements

After overnight fasting blood glucose was measured using a standard glucose meter (FreeStyle Precision Neo Meter, Abbott, Chicago, USA), and a small amount of venous blood was collected from the tail for the measurement of plasma insulin concentration using an ELISA kit (Mercodia, Uppsala, Sweden). Afterwards, the oGTT was performed with an oral gavage of 2 g/kg of glucose. Blood was sampled from the tip of the tail at 0, 15, 30, 60, 90, 120 min. The Homeostatic Model Assessment of Insulin Resistance (HOMA-IR) was calculated according to the Eq 1.


HOMA−IR=(fastingglucose[mmol/l].fastinginsulin[mIU/l])/22.5 (1)

Quantitative Insulin Sensitivity Check Index (QUICKI) was calculated according to the Eq 2.


QUICKI=1/((fastinginsulin[mg/l]+(fastingglucose[mmol/l])) (2)

Total area under the curve (AUC) of the glucose dynamics from oGTT was calculated for each individual. Incremental AUC was calculated as AUC over the basal fasting glucose.

### Sacrifice and organ processing

At the end of experimental procedures, the mice were anesthetized with isoflurane (Piramal Healthcare, London, UK). Blood was collected from the retroorbital sinus. Immediately after blood collection, liver was collected, weighted and snap-frozen using liquid nitrogen and stored at -80°C for further analyzes. Liver tissue (100 mg) was homogenized and extracted in chloroform/methanol according to the protocol by Jover et al. [22]. for determination of triacylglycerols concentrations in the liver. Similarly, 100 mg of liver was used for cholesterol determination in the liver according to modified protocol by Abel et al. [23].

### Quantification of ecDNA concentration

Blood was collected into both, EDTA- and heparin-containing tubes and centrifuged at 1,600g for 10 min at 4°C. EDTA plasma was centrifuged again at 16,000g for 10 min at 4°C. The supernatant was stored until analysis at -20°C. EDTA plasma was used for isolation of DNA using the QIAamp DNA Blood Mini kit (Qiagen, Hilden, Germany). Concentration of ecDNA was measured using a fluorometric method with Qubit 3.0 fluorometer and Qubit dsDNA HS assay (Thermo Fisher Scientific, Waltham, MA, USA).

### DNase activity measurement

DNase activity was measured using the modified single radial enzyme diffusion assay in heparin plasma [24]. Briefly, agarose gels were prepared with calcium chloride (2 mM), magnesium chloride (2 mM) and DNA isolated from chicken liver (0.5 mg/ml). Heparin plasma samples were applied and after 18-hours of incubation, pictures of gels were made using iBOX (Vision works LP Analysis Software, UVP, Upland, CA, USA). Diameter of circles on gel was measured in ImageJ software (NIH, Maryland, USA).

### Statistical analysis

The statistical analysis was performed using GraphPad software 8.0.2 (GraphPad Software, Inc., San Diego, CA, USA). Pearson´s correlation analysis was used to evaluate the association between ecDNA or DNase activity with fasting glucose and insulin concentrations in plasma, or total AUC, incremental AUC, HOMA-IR and QUICKI. P-values below 0.05 were considered as statistically significant.

## Results

### Correlation between ecDNA/DNase activity and body weight or liver parameters

**Fig 1:**
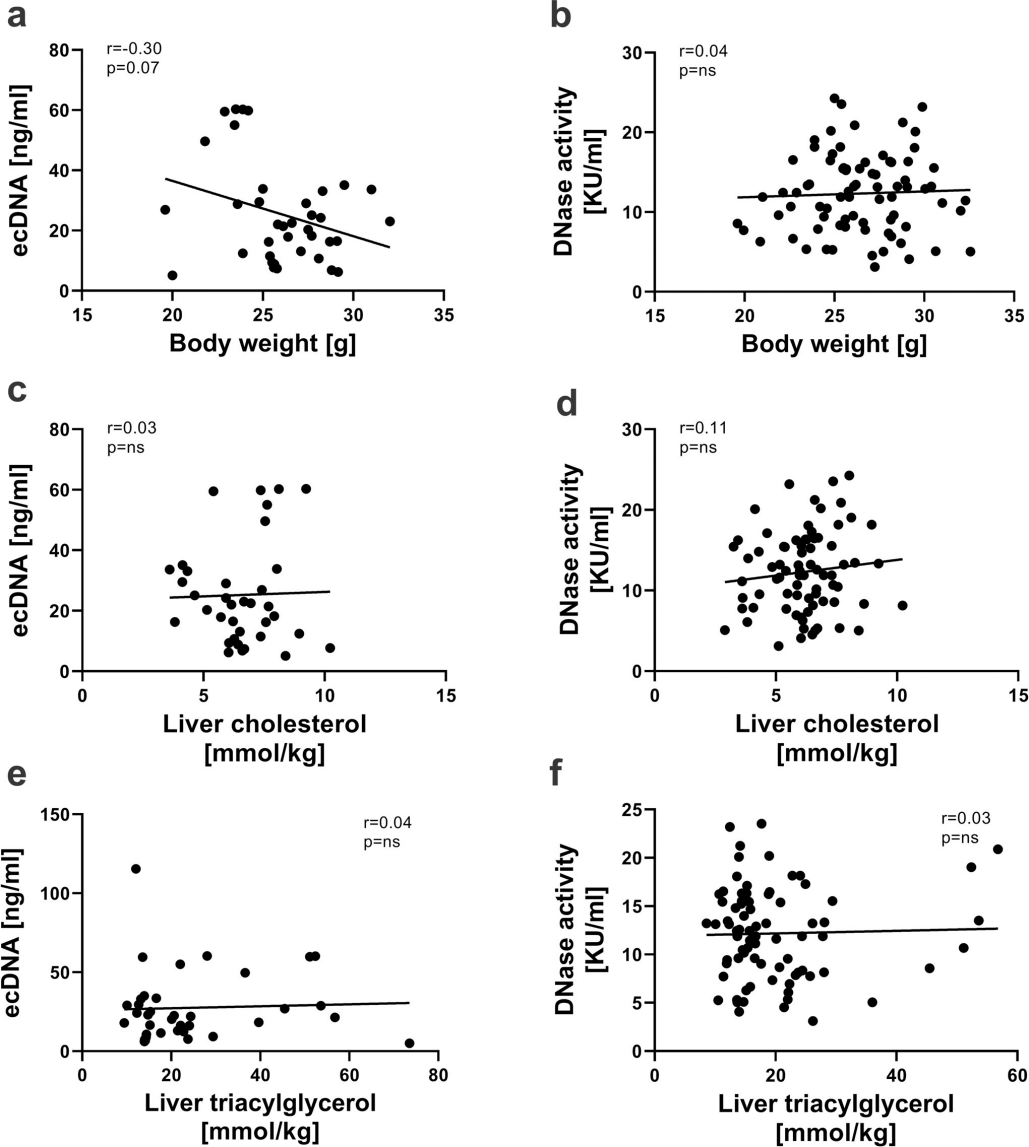
Relationship of ecDNA and DNase activity with body weight, cholesterol and triacylglycerol in the liver. Correlations between ecDNA and body weight (a), cholesterol (c) or triacylglycerol in the liver (e). Correlations between DNase activity and body weight (b), cholesterol (d) or triacylglycerol in the liver (f). ecDNA—extracellular DNA; KU—Kunitz units.

Neither correlations between ecDNA and body weight (r = -0.30, p = 0.068, Fig 1A), cholesterol (r = 0.03, p = 0.876, Fig 1C) or triacylglycerol (r = 0.04, p = 0.798, Fig 1E), nor correlations between DNase activity and body weight (r = 0.04, p = 0.718, Fig 1B), cholesterol (r = 0.11, p = 0.324, Fig 1D) or triacylglycerol in the liver (r = 0.03, p = 0.806, Fig 1F) were found.

### Correlation between ecDNA/DNase activity and fasting glycemia or area under the curve (AUC) of glycemia during oGTT

**Fig 2:**
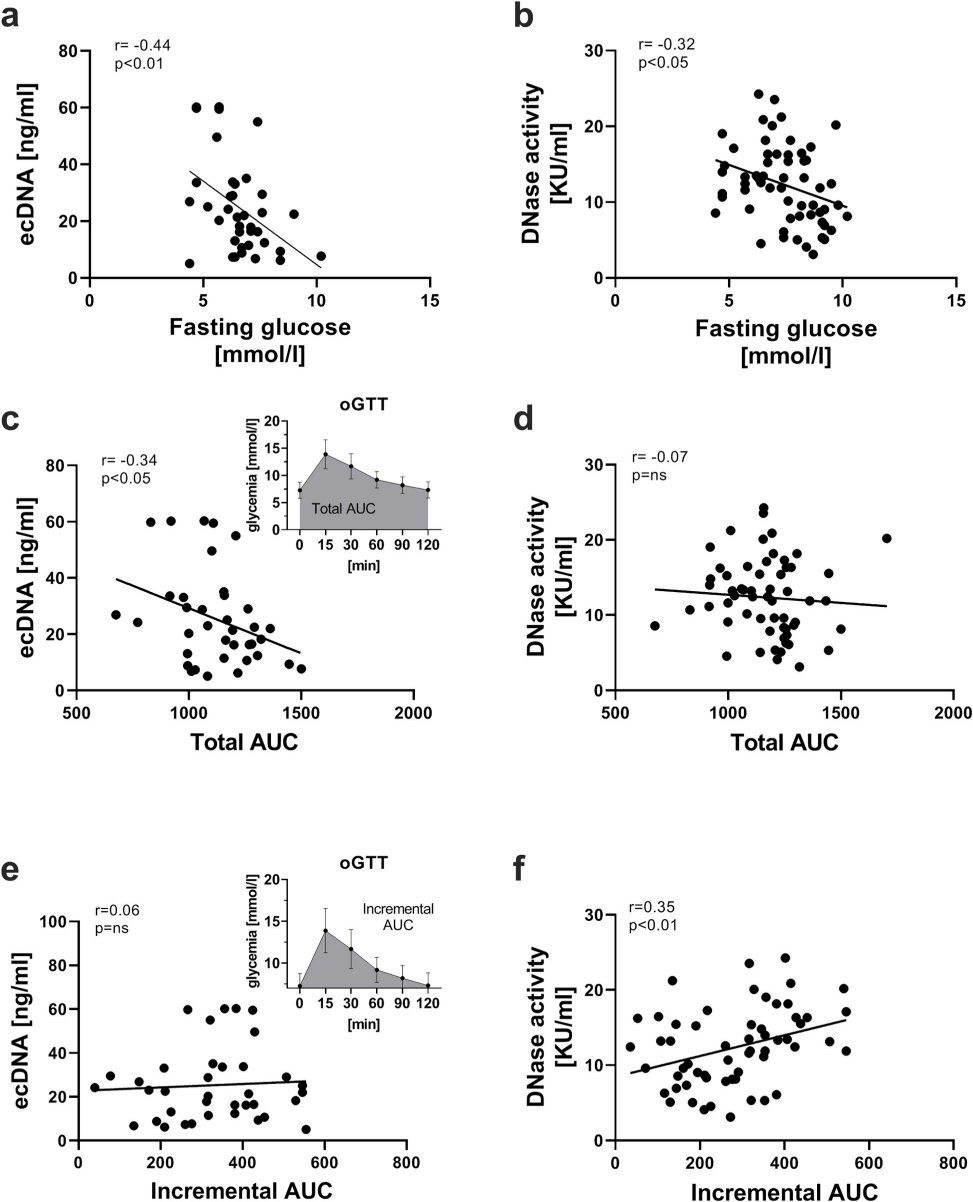
Relationship of ecDNA and DNase activity with fasting glucose, total and incremental AUC. Correlations between ecDNA and fasting glucose (a), total AUC (c) or incremental AUC (e). Correlations between DNase activity and fasting glucose (b), total AUC (d) or incremental AUC (f). ecDNA—extracellular DNA; AUC—area under the curve; KU—Kunitz units.

Significant negative correlations between ecDNA and fasting glucose (r = -0.44, p = 0.006, Fig 2A), DNase activity and fasting glucose (r = -0.32, p = 0.015, Fig 2B), as well as ecDNA and total AUC (r = -0.34, p = 0.039, Fig 2C) have been observed. Higher DNase activity was associated with higher incremental AUC (r = 0.35, p = 0.007, Fig 2F). Correlations were found neither between ecDNA and incremental AUC (r = 0.06, p = 0.717, Fig 2E), nor between DNase activity and total AUC (r = -0.07 p = 0.584, Fig 2D).

### Correlations between ecDNA/DNase activity and insulin sensitivity

**Fig 3:**
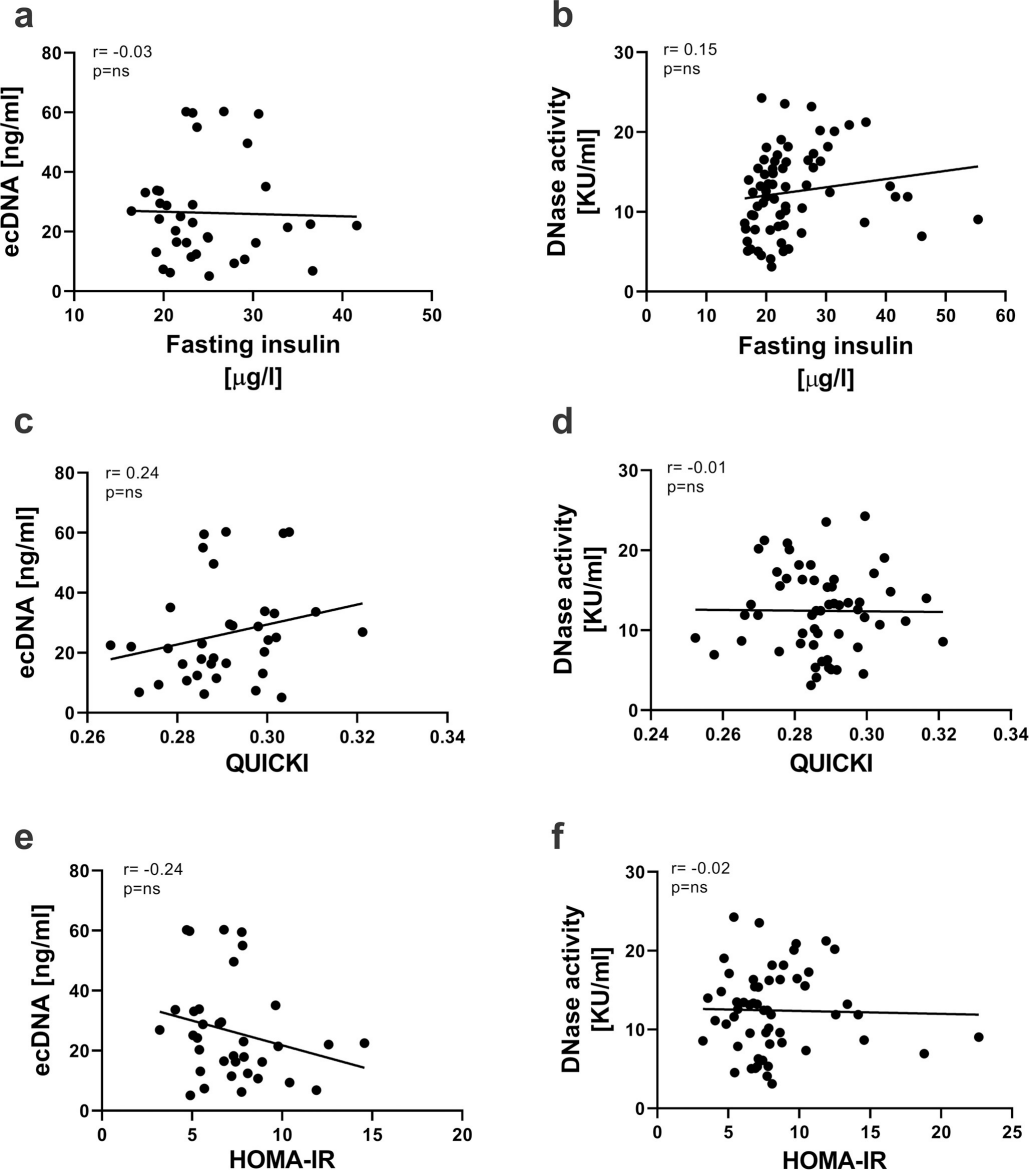
Relationship of ecDNA and DNase activity with fasting insulin, QUICKI and HOMA-IR. Correlations between ecDNA and fasting insulin (a), QUICKI (c) or HOMA-IR (e). Correlations between DNase activity and fasting insulin (b), QUICKI (d) or HOMA-IR (f). ecDNA. ecDNA—extracellular DNA; HOMA-IR—Homeostatic Model Assessment of Insulin Resistance; QUICKI -Quantitative Insulin Sensitivity Check Index; KU—Kunitz units.

Significant correlations were found neither between ecDNA and fasting insulin (r = -0.03, p = 0.878, Fig 3A), QUICKI (r = 0.24, p = 0.167, Fig 3C) or HOMA-IR (r = -0.24, p = 0.158, Fig 3E), nor between DNase activity and fasting insulin (r = 0.15, p = 0.214, Fig 3B), QUICKI (r = -0.01, p = 0.938, Fig 3D) or HOMA-IR (r = -0.02, p = 0.855, Fig 3F).

## Discussion

The results of our cross-sectional study on adult female mice show that ecDNA is not related to bodyweight, but high ecDNA is associated with lower fasting glycemia and total AUC in oGTT. These results are in contrast to our expectations. Previous studies in humans showed that obesity is associated with higher plasma ecDNA concentrations [12,14]. However, in our experiment the bodyweight of the mice varied within physiological variability. The association ecDNA and bodyweight might be different in healthy mice and in mice with induced obesity. Also, the situation might be different in induced obesity models while mice are gaining weight and, in our study, where the bodyweight of the animals was relatively stable. One of the major limitations of our study is that the cross-sectional design does not allow observations of dynamics changes. On the other hand, analysis of physiological variability is an important cornerstone of the pathophysiology of incident obesity.

Lower fasting glycemia and total AUC associated with higher ecDNA suggest that a better glucose tolerance is higher concentrations of a cell-damage marker. This is counterintuitive and surprising in light of the previously published data. Although the effect size is rather small the observed negative correlations should be further examined. Associations do not have to be causal and in this case, not even the direction of the potential causality is clear. In our study we have not analyzed the physical activity of the mice, nor the stress level. Both, however, were shown to affect the plasma ecDNA [25–27]. Physical activity, but also stress via endocrine effects could influence the glucose metabolism [28,29]. It is possible that mice with higher locomotor activity have a better glucose metabolism, but also more ecDNA in plasma due to the physical activity. However, glucose metabolism could also affect the release or the degradation of ecDNA.

DNase activity in plasma correlated negatively with fasting glucose and positively with incremental AUC in oGTT. These findings seem to be contradictory, but the incremental AUC is influenced by fasting glycemia–the higher the fasting glycemia, the lower the incremental AUC in oGTT. We, thus, cannot exclude the possibility that the association with incremental AUC is secondary. In our previous study ecDNA and DNase in plasma did not correlate [30]. This suggests that besides nucleases other mechanisms are involved in ecDNA degradation, but the lack of an association could also be explained by the healthy status of the mice. The administration of exogenous DNase decreases plasma ecDNA as shown in several of our previous experiments [10,11]. Whether the physiological inter- or intraindividual variability of DNase has any effect on ecDNA and its immunogenicity is unknown. The extremely short half-life of fetal DNA in maternal circulation after delivery, however, suggests that the cleavage of ecDNA is very efficient and, thus, likely important [6,31].

Other mechanisms of ecDNA cleavage besides DNases have already been postulated, but not yet uncovered [32]. Liver could clean blood plasm from ecDNA, as liver damage leads to an increase of ecDNA in plasma [33]. However, it is not clear whether this increase is due to ecDNA release from damaged hepatocytes or from the decrease in ecDNA cleavage by the liver. We have analyzed the liver lipids as a proxy of liver damage. None of the healthy mice had steatosis and the observed physiological variability of liver lipids had no association with ecDNA or DNase. Interestingly, neither liver lipids nor ecDNA or DNase were associated with insulin sensitivity in our study on healthy adult female mice. Of course, we cannot extrapolate our findings on adolescent or aged mice. Similarly, interpretation of our results is limited by the fact that we have not included postmenopausal mice. Glucose metabolism is affected by several endocrine factors other than insulin and insulin sensitivity [34]. Neither glucagon, nor incretins were assessed in this study, but potentially could modulate the relationship between ecDNA, DNase and glucose metabolism.

A cross-sectional study as a study design is rarely used in mice. We have decided to study the potential associations in this way, because CD1 mice are outbred and so their genetic variability is more similar to the variability in humans, at least in comparison to the widely used inbred strains [35]. Interventional experiments have their strengths, but they often do not reproduce the biological variability in humans and, thus, they are difficult to interpret with regards to the physiological variance. A major limitation of our study is that we have only focused on glucose metabolism and did not analyze the presence of other components of the metabolic syndrome. Thus, we have not studied a disease or a disease model. The results should surely not be interpreted with regards to metabolic pathologies. Similarly, we have not tested the subcellular or tissue origin of the plasma ecDNA using real time or methylation-sensitive PCR, respectively. On the other hand, our study is the first to study plasma ecDNA in relation to glucose metabolism in healthy mice. In addition, this experiment is the first to study the metabolic associations of the biological variability of ecDNA and DNase. As some sex differences in DNase have been reported in the past, it would surely be interested to analyze the same associations in male mice as well [30].

In summary, we have shown for the first time that low ecDNA in healthy adult female mice is associated with higher fasting glucose and lower glucose tolerance. In addition, the results indicate that low endogenous DNase studied for the first time in relation to metabolic parameters is associated with higher fasting glucose. The causality of the observed associations should be reproduced in other experimental animals and healthy human probands, and ideally proved, and their direction further studied in interventional experiments. Especially, as novel therapeutic targets for prediabetes and metabolic syndrome are highly needed.

## Supporting information

S1 Dataset Table of data used in this manuscript. (XLSX)
